# 403. Deep Learning–Based Prediction of Antimicrobial Resistance Using Multicenter Matrix-Assisted Laser Desorption/Ionization–Time-of-Flight (MALDI-TOF) Mass Spectrometry Data

**DOI:** 10.1093/ofid/ofaf695.141

**Published:** 2026-01-11

**Authors:** Yu-Chun Pan, Shu-Yu Tsao, Yueh-Chen Hsieh, Chih-Hung Wang, Matthew Huei-Ming Ma, Wang-Huei Sheng, Po-Chun Liao, Chien-Chang Lee

**Affiliations:** National Taiwan University College of Medicine, Taipei, Taipei, Taiwan (Republic of China); Department of Emergency Medicine, National Taiwan University Hospital, Taipei, Taiwan., Taipei, Taipei, Taiwan; National Taiwan University Hospital Yunlin Branch, Taiwan, Yunlin County, Yunlin, Taiwan; National Taiwan University Hospital, Taipei, Taipei, Taiwan; National Taiwan University Hospital Yunlin Branch, Yunlin, Yunlin, Taiwan; National Taiwan University Hospital, Taipei, Taipei, Taiwan; National Taiwan University, Taipei, Taipei, Taiwan; National Taiwan University; Ministry of Health and Welfare, Taiwan, Taipei, Taipei, Taiwan

## Abstract

**Background:**

Sepsis is a leading cause of death, and early identification of pathogens with appropriate antibiotics improves survival. Antimicrobial resistance (AMR) complicates empirical therapy, while conventional diagnostics take 48–72 hours. Although MALDI-TOF mass spectrometry can identify pathogens within 24 hours, it cannot predict resistance. Prior machine learning models on spectra data showed only moderate performance (area under the receiver operating characteristic curve [AUROC] ≈ 0.70), limiting clinical utility.

Representative Antibiotic-Resistance Predictions for 26 Bacterial Species

**Figure:**
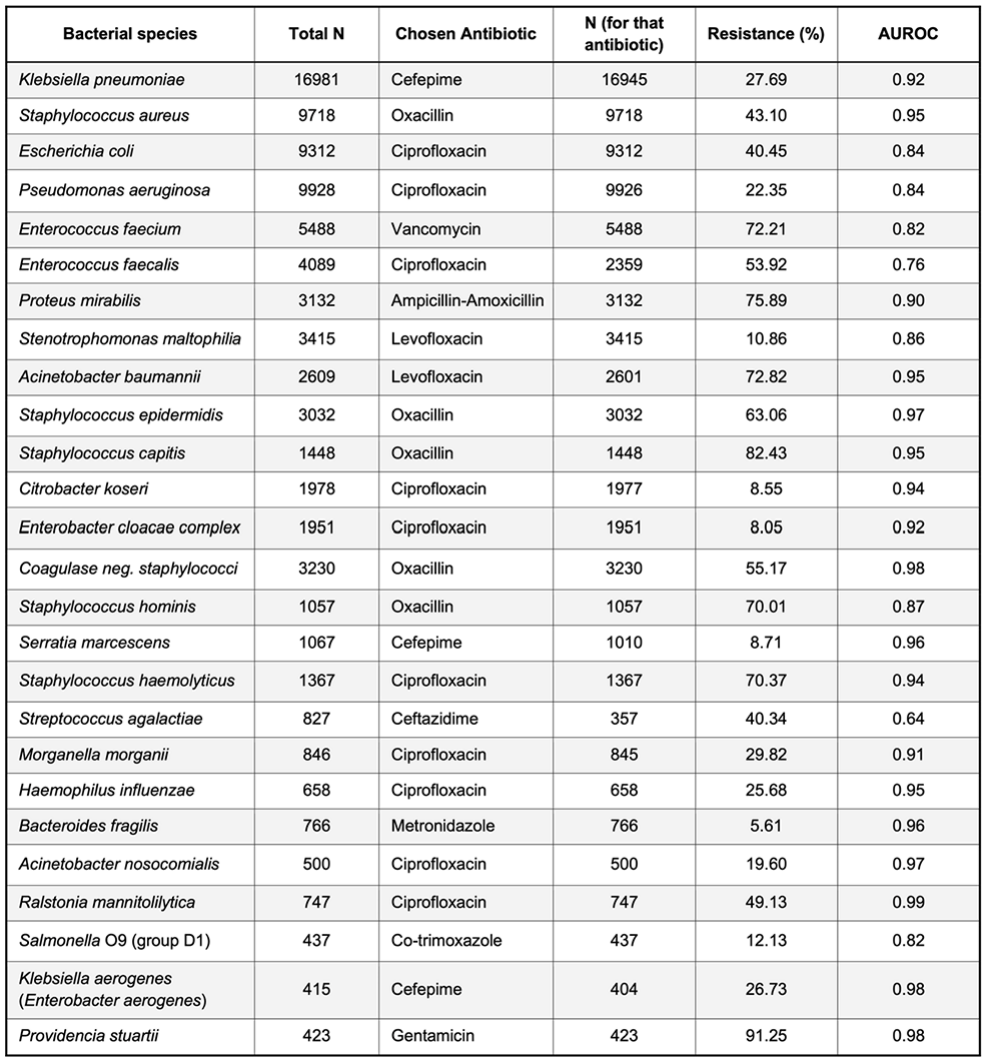

This table summarizes the best-performing antibiotic-resistance predictions for each of the 26 bacterial species in our dataset. For each species, we selected the antibiotic that achieved the highest AUROC or held particular clinical relevance (e.g., widely used empirically or known to have critical resistance patterns). The “Total N” column indicates the overall sample size for that species; “N (for that antibiotic)” shows how many samples were tested for that specific drug-resistance pair. The AUROC reflects our TCN-Transformer model’s predictive accuracy (1.00 = perfect discrimination). We selected antibiotics with moderate-to-high clinical relevance, non-trivial resistance prevalence, and robust AUROC performance to highlight the model’s true discriminative capacity.

**Figure:**
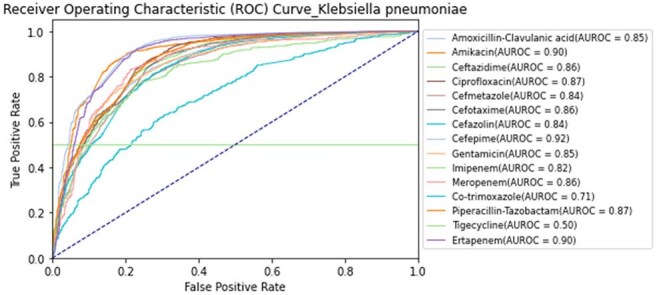
AUROC Curves for Antimicrobial Resistance Predictions in Klebsiella pneumoniae Each curve represents the TCN–Transformer model’s performance in predicting resistance to a specific antibiotic.

**Methods:**

We developed deep learning models using temporal convolutional networks (TCNs) combined with Transformer layers to predict AMR from MALDI-TOF spectra. Data from 26 bacterial species were collected from two hospitals in Taiwan and Switzerland. Preprocessing included binning, variance stabilization, SNIP baseline correction, and total ion current normalization. Resistance was labeled using European Committee on Antimicrobial Susceptibility Testing (EUCAST) and Clinical and Laboratory Standards Institute (CLSI) breakpoints. AUROC was the primary performance metric. For each species, we reported the antibiotic with the most clinically and statistically meaningful prediction based on resistance prevalence and AUROC. AI/LLM disclosure – GPT-4 was used solely for language editing and formatting; all content was written and approved by the authors.

**Figure:**
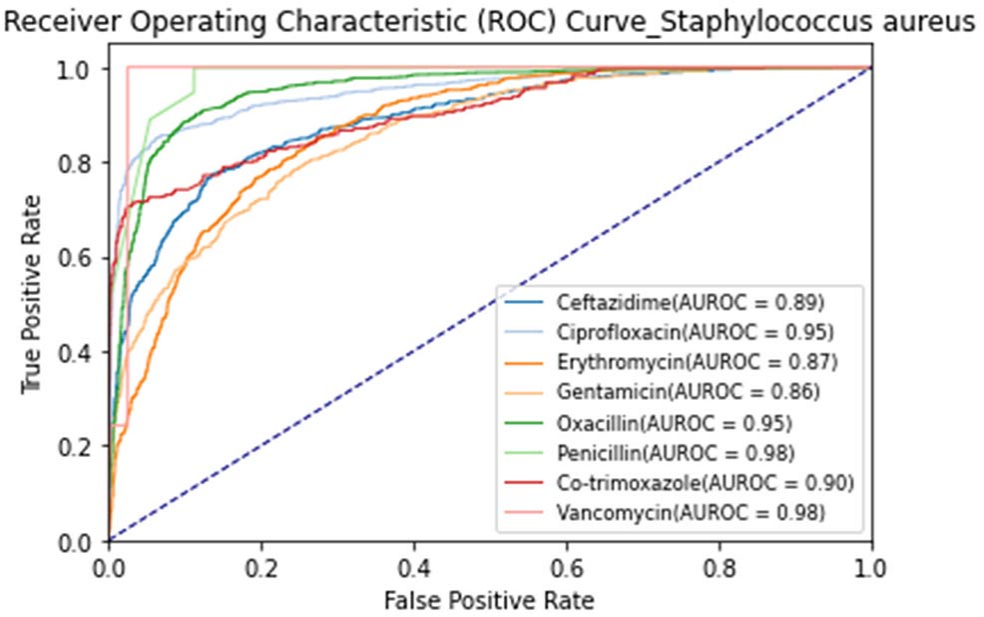
AUROC Curves for Antimicrobial Resistance Predictions in Staphylococcus aureus Each curve represents the TCN–Transformer model’s performance in predicting resistance to a specific antibiotic.

**Figure:**
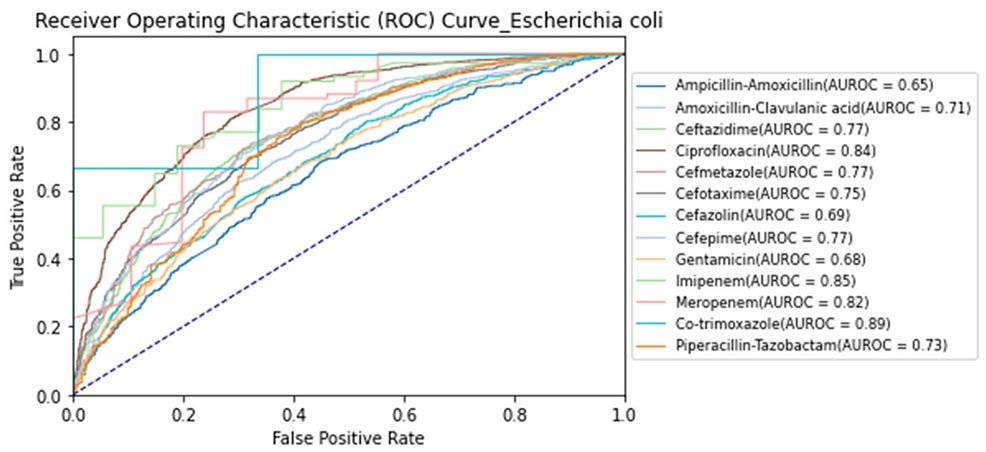
AUROC Curves for Antimicrobial Resistance Predictions in Escherichia coli Each curve represents the TCN–Transformer model’s performance in predicting resistance to a specific antibiotic.

**Results:**

We analyzed 85,421 MALDI-TOF spectra from 26 bacterial species and selected the best-performing antibiotic-resistance pair per species. Of these pairs, 22 achieved AUROCs ≥ 0.84. Notable results included AUROCs of 0.92 for *Klebsiella pneumoniae* (cefepime), 0.95 for *Staphylococcus aureus* (oxacillin), and 0.84 for *Escherichia coli* and *Pseudomonas aeruginosa* (ciprofloxacin). *Acinetobacter baumannii* (levofloxacin) and *Staphylococcus epidermidis* (oxacillin) both exceeded 0.95. Performance was consistent across institutions, supporting the generalizability of TCN–Transformer models for early, accurate AMR prediction.

**Conclusion:**

Deep learning applied to MALDI-TOF spectra enables rapid, accurate AMR prediction across diverse pathogens. Our TCN-Transformer models markedly outperform prior models and may support earlier, more precise antibiotic therapy.

**Disclosures:**

All Authors: No reported disclosures

